# Dynamic risk stratification and treatment optimization in sepsis: the role of NLPR

**DOI:** 10.3389/fphar.2025.1572677

**Published:** 2025-04-02

**Authors:** Qiqi Chen, Ming Zhang, Yuxin Xia, Ya Deng, Yanna Yang, Lili Dai, Hongxia Niu

**Affiliations:** ^1^ Department of Emergency, Capital Medical University Electric Power Teaching Hospital (State Grid Corporation of China Beijing Electric Power Hospital), Beijing, China; ^2^ Department of Cardiovascular Medicine, Capital Medical University Electric Power Teaching Hospital (State Grid Corporation of China Beijing Electric Power Hospital), Beijing, China

**Keywords:** sepsis, NlpR, prognostic biomarker, MIMIC-IV, mortality, risk stratification

## Abstract

**Background:**

Sepsis, characterized by immune dysregulation, inflammatory cascades, and coagulation dysfunction, remains a global health challenge with high mortality, particularly in patients with multiple organ dysfunction syndrome (MODS). Existing prognostic tools, such as SOFA and APACHE II scores, are limited by complexity and lack of real-time monitoring, necessitating simple and reliable biomarkers for risk stratification and individualized management.

**Objective:**

This study aimed to evaluate the prognostic value of the neutrophil-to-lymphocyte-to-platelet ratio (NLPR) for mortality in sepsis patients and explore its potential utility in dynamic risk stratification and treatment optimization.

**Methods:**

We conducted a retrospective cohort study using the MIMIC-IV database (v3.1), including adult sepsis patients meeting Sepsis-3.0 criteria. NLPR was calculated based on neutrophil, lymphocyte, and platelet counts within 24 h of admission. Patients were stratified into quartiles (Q1-Q4) based on NLPR values. Kaplan-Meier survival analysis, Cox regression models, and restricted cubic spline (RCS) analysis were performed to assess NLPR’s association with 28-day, 90-day, and 365-day mortality. Subgroup analyses examined NLPR’s performance in diverse clinical populations.

**Results:**

NLPR was a strong and independent predictor of mortality at all time points. Patients in the highest NLPR quartile (Q4) had significantly higher 28-day (28.22% vs. 12.64%), 90-day (36.82% vs. 18.06%), and 365-day (44.94% vs. 25.58%) mortality compared to the lowest quartile (Q1, all P < 0.001). Cox regression confirmed the independent association of high NLPR with mortality after adjusting for confounders such as age, gender, BMI, and SOFA scores. RCS analysis identified nonlinear relationships between NLPR and mortality, with critical thresholds (e.g.,NLPR = 6.5 for 365-day mortality) providing actionable targets for early risk identification. Subgroup analysis revealed consistent predictive performance across clinical populations, with amplified risks in younger patients, malnourished individuals, and those with acute kidney injury.

**Conclusion:**

NLPR is a simple, accessible, and robust biomarker for sepsis risk stratification, integrating inflammation and coagulation data. It complements traditional scoring systems, provides actionable thresholds for early intervention, and facilitates dynamic monitoring. These findings underscore NLPR’s potential to improve clinical decision-making and outcomes in sepsis management, warranting validation in prospective multicenter studies.

## 1 Introduction

Sepsis is a systemic inflammatory response syndrome (SIRS) triggered by infection. It is characterized by immune dysregulation, organ dysfunction, and a high mortality rate associated with infection, contributing significantly to global morbidity and mortality (Fleischmann-Struzek and Rudd, 2023; Kelly et al., 2016; Moya et al., 2020). The Global Burden of Disease Study reported approximately 49 million new sepsis cases annually, with the incidence rate in developing countries being significantly higher than in developed nations (Murray, 2022). In developed countries, the annual incidence rate of sepsis ranges from 276 to 678 cases per 100,000 population, and sepsis accounts for approximately 20% of total global deaths (Weng et al., 2023). The in-hospital mortality rate for patients with sepsis is estimated to be between 22.5% and 26.7%, with this risk further elevated in patients with multiple organ dysfunction syndrome (MODS) (Fleischmann et al., 2016).

Despite considerable advancements in anti-infective treatments, intensive care, and organ support technologies, the mortality rate associated with sepsis remains alarmingly high. The complex pathophysiological mechanisms underlying sepsis include inflammatory dysregulation, immunosuppression, coagulation disorders, and microcirculatory impairment, all of which complicate the accurate prediction of patient prognosis (Weng et al., 2023). Although commonly used clinical scoring systems, such as the SOFA score and APACHE II score, provide some assistance in assessing sepsis risk, their utility in rapid decision-making is limited by complex calculations and challenges in dynamic monitoring (Fleischmann-Struzek and Rudd, 2023). Consequently, there is an urgent need for reliable biomarkers to evaluate the severity, progression, and early intervention effects of sepsis to enhance patient management and improve prognosis.

In recent years, hematological markers have garnered increasing attention due to their convenience, low cost, and capacity to dynamically reflect inflammation and coagulation status. These markers include C-reactive protein (CRP), procalcitonin (PCT), lactate, soluble urokinase plasminogen activator receptor (suPAR), mean platelet volume (MPV), and adrenomedullin (pro-adrenomedullin), all of which have been utilized for the prognostic assessment of sepsis. Among these, the neutrophil-to-lymphocyte ratio (NLR) and the platelet-to-lymphocyte ratio (PLR) have emerged as significant research focuses due to their potential to indicate dynamic responses to inflammation and coagulation (Jayara et al., 2024; Leroux et al., 2021; Zhang et al., 2022). However, the predictive ability of individual markers may be constrained by unilateral changes, complicating the comprehensive characterization of sepsis, a multifaceted condition that involves multiple systems.

In this context, NLPR has been proposed as an emerging comprehensive hematological marker (Martinez et al., 2024). NLPR integrates data on inflammation and coagulation, providing a more holistic reflection of the pathophysiological status in patients with sepsis. Research indicates that elevated NLPR values may signify a severe inflammatory response and immune suppression, thereby increasing the risk of organ failure and mortality (Su et al., 2023). Concurrently, a low platelet count may serve as an early indicator of microvascular disease and disseminated intravascular coagulation (DIC), further underscoring the clinical relevance of NLPR (Wang et al., 2024). Although preliminary studies have demonstrated a significant association between high NLPR values and poor prognosis in sepsis patients, the findings remain inconsistent due to variations in sample size, patient characteristics, and statistical methodologies (Pantea et al., 2024). Moreover, the optimal predictive threshold for NLPR and its applicability across different patient subgroups warrant further investigation.

This study aimed to systematically evaluate the predictive value of the NLPR in patients with sepsis. By analyzing a large sample of retrospective data, we investigated the relationship between NLPR and all-cause mortality at 28, 90, and 365 days. We employed a combination of Kaplan-Meier survival analysis and Cox regression models to confirm the independent predictive role of NLPR. Additionally, we explored the nonlinear association between NLPR and mortality using the restricted cubic spline method to identify potential threshold effects. The findings of this study are anticipated to provide new predictive tools for clinical application and to enhance risk stratification and individualized treatment strategies for patients with sepsis.

## 2 Methodology

### 2.1 Study design and data source

This study was a retrospective cohort analysis using data from the Medical Information Mart for Intensive Care IV (MIMIC-IV, version 3.1). The MIMIC-IV database is a large, publicly accessible collection of clinical information from patients treated in intensive care units (ICUs) at Beth Israel Deaconess Medical Center (BIDMC). Before use, the database was approved by ethics committees at the Massachusetts Institute of Technology (MIT) and BIDMC. Additionally, all patient information was anonymized in accordance with Health Insurance Portability and Accountability Act (HIPAA) guidelines. Therefore, no further ethics approval was required for our study.

### 2.2 Study population

Inclusion criteria encompassed adult patients who fulfilled the diagnostic criteria for sepsis according to the Sepsis-3.0 guidelines. The exclusion criteria were as follows:

(1) Missing key laboratory data within 24 h of admission, including neutrophil count, lymphocyte count, and platelet count;
(2) Patients younger than 18 years or older than 90 years;
(3) Duplicate admission records;
(4) Patients who died within 48 h of admission;
(5) Patients with conditions potentially affecting NLPR values significantly, such as hematological malignancies.

**FIGURE 1:**
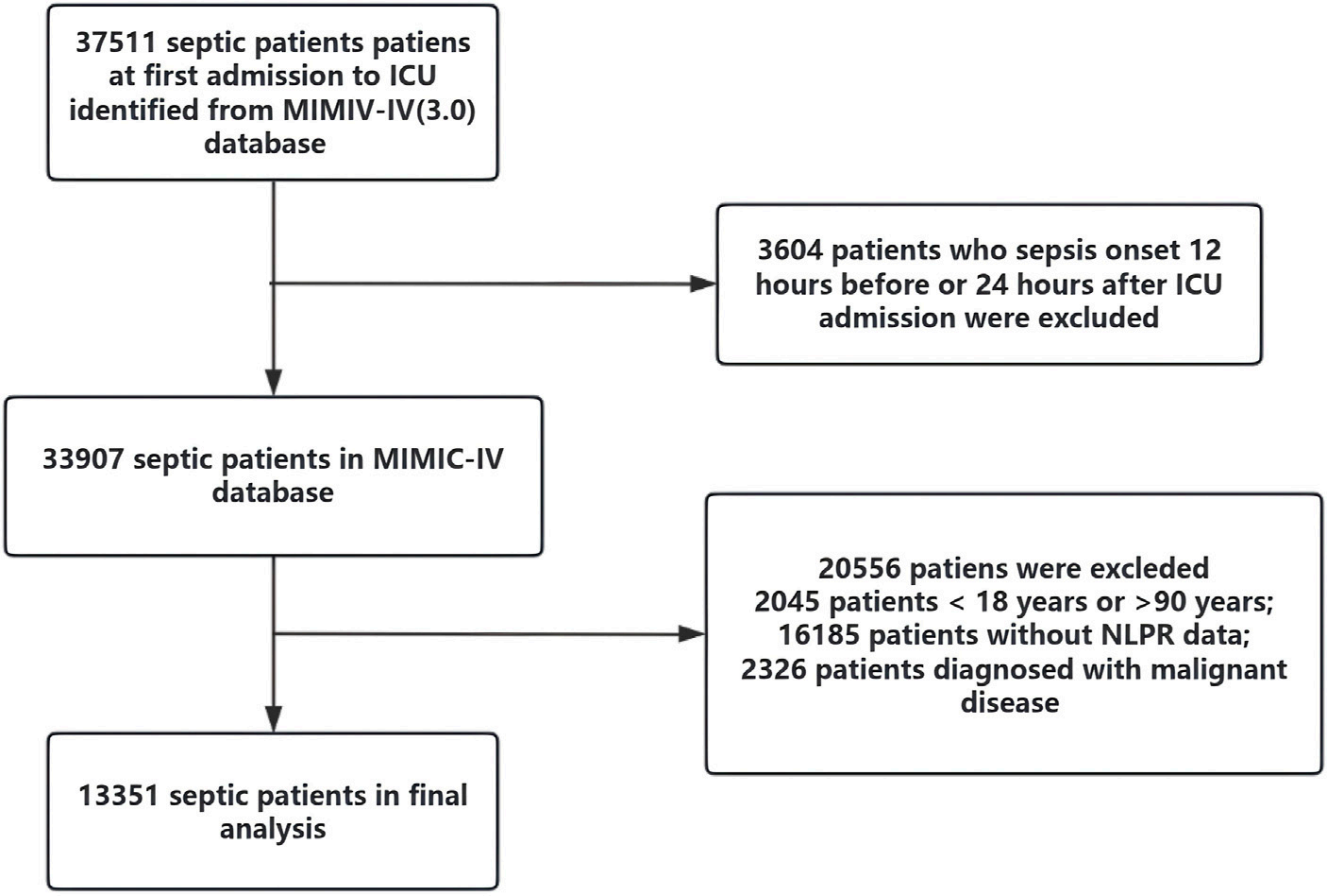
The flow chart of inclusion and exclusion.

Patients were categorized into four quartiles (Q1–Q4) based on their NLPR values at admission to explore the relationship between NLPR and clinical outcomes. The patient selection process is outlined in Figure 1. After applying these criteria, the final cohort consisted of 13,351 patients with sepsis.

### 2.3 Outcomes

The primary outcome was 1-year all-cause mortality. The secondary outcomes included 28-day and 90-day all-cause mortality.

### 2.4 Variables and parameters

NLPR was calculated as $\frac{\text{neutrophil}\;\text{count}\times 100}{\text{lymphocyte}\;\text{count}\times \text{platelet}\;\text{count}}$ .Patients were categorized into four groups based on the quartiles of NLPR (Q1–Q4): Low group (Q1): NLPR <2.62 (25th percentile); Intermediate group (Q2 and Q3): 2.62 (25th percentile) ≤ Q2 < 4.80 (50th percentile) and 4.80 (50th percentile) ≤ Q3 < 9.67 (75th percentile); High group (Q4): NLPR ≥9.67 (75th percentile).

The following clinical and laboratory variables were comprehensively extracted from the MIMIC-IV database for evaluating patient demographics, comorbidities, inflammatory and coagulation status, organ dysfunction, disease severity, and therapeutic interventions:

(1) Demographic Characteristics:Age, gender, body mass index (BMI), and marital status.
(2) Comorbidities:
  1) Hypertension, diabetes mellitus, coronary heart disease, chronic obstructive pulmonary disease (COPD), stroke, chronic kidney disease, chronic liver disease, malignancy, and other relevant conditions;
  2) Charlson Comorbidity Index, calculated based on the presence of predefined chronic conditions, was included to quantify comorbidity burden.
(3) Laboratory Parameters
  1) Inflammatory Markers:WBC,neutrophil count, lymphocyte count,NLR, PCT, and CRP.
  2) Coagulation Markers: PLT, prothrombin time, and D-dimer.
  3) Organ Dysfunction Markers:

Renal Function Indicators: Serum creatinine, blood urea nitrogen; Hepatic Function Indicators: Total bilirubin, ALT, AST, and serum albumin; Metabolic and Oxygenation Markers: Lactate, PaO_2_, FiO_2_, PaO_2_/FiO_2_ ratio, bicarbonate (HCO_3_^−^), and arterial pH.

These laboratory parameters were specifically chosen based on their inclusion as essential components in established severity scoring systems such as APSIII, SAPS II, SOFA, LODS, and MELD.

(4) Disease Severity and Organ Dysfunction Scores:

The following disease severity and prognostic scores were calculated, incorporating clinical parameters (e.g., heart rate, respiratory rate, mean arterial pressure, temperature), laboratory indicators (e.g., lactate, creatinine, bilirubin, platelet count, PaO_2_/FiO_2_ ratio), and neurological assessments (Glasgow Coma Scale, GCS).

APSIII, SAPS II, and OASIS: vital signs, PaO_2_/FiO_2_ ratio, renal function indicators (creatinine and BUN), hepatic function markers (bilirubin, ALT, AST), hematological parameters (platelets, WBC count), coagulation parameters (prothrombin time), electrolytes, and neurological status (Glasgow Coma Scale).

SOFA and LODS: Creatinine, bilirubin, platelets, coagulation tests (PT), respiratory function (PaO2/FiO2 ratio), neurological assessments (GCS), and vasoactive medication usage.

MELD: Serum bilirubin, serum creatinine, international normalized ratio (INR), and sodium levels.

All relevant parameters used for calculating these scores were explicitly recorded and analyzed to ensure methodological rigor and reproducibility.

### 2.5 Statistical

Analysis was performed using R software (v4.2.0). Continuous variables were described as medians with interquartile ranges (IQR) and compared across groups using the Kruskal–Wallis test, while categorical variables were presented as frequencies and percentages, with group differences assessed via the chi-square test. Kaplan-Meier survival curves were utilized to depict survival probabilities, and the Log-rank test was applied to compare survival outcomes among NLPR quartiles. The relationship between NLPR and all-cause mortality at 28, 90, and 365 days was analyzed using Cox proportional hazards regression models, adjusting for confounders such as age, gender, BMI, and SOFA score, with results expressed as hazard ratios and 95% confidence intervals. To investigate nonlinear associations between NLPR and 365-day mortality, the Restricted Cubic Spline (RCS) method was employed, identifying critical value thresholds. Subgroup analyses were conducted to evaluate the predictive performance of NLPR within specific strata (e.g., age, gender, BMI, SOFA score) and to explore potential interactions with subgroup variables. Multiple imputation techniques were used to address missing data, ensuring robust and unbiased results. A two-sided P value <0.05 was considered statistically significant.

## 3 Results

### 3.1 General characteristics of the study cohort

The baseline characteristics of patients differed significantly across the four NLPR quartiles (P < 0.05), indicating substantial clinical heterogeneity (Table 1). Patients in the higher NLPR quartiles (Q3 and Q4) were older (P < 0.001) and had a higher proportion of males (P < 0.001). Socioeconomic factors may also contribute to these differences, as a greater proportion of single and widowed individuals were observed in Q4 (P = 0.002), potentially influencing healthcare access, disease progression, and long-term prognosis.

**TABLE 1:** Comparison of general characteristics across four patient groups.

Variables	NLPR (P25, P75)				Test value	*p*
	Q1 (*n* = 3,338)	Q2 (*n* = 3,337)	Q3 (*n* = 3,338)	Q4 (*n* = 3,338)		
Age (years)	65.337 (53.7,75.4)	66.561 (56.0,76.3)	66.940 (55.7,76.8)	65.889 (54.6,76.2)	23.00	<0.001
Gender, n (%)					χ^2^ = 18.08	<0.001
Male	1854 (55.54)	1994 (59.75)	1989 (59.59)	1997 (59.83)		
Female	1484 (44.46)	1343 (40.25)	1349 (40.41)	1341 (40.17)		
Marital status, n (%)					χ^2^ = 25.97	0.002
Single	998 (32.74)	930 (31.09)	897 (30.21)	987 (33.51)		
Married	1424 (46.72)	1496 (50.02)	1491 (50.22)	1389 (47.16)		
Divorced	303 (9.94)	232 (7.76)	233 (7.85)	258 (8.76)		
Widowed	323 (10.60)	333 (11.13)	348 (11.72)	311 (10.56)		
Smoker, n (%)	224 (6.71)	240 (7.19)	229 (6.86)	217 (6.50)	χ^2^ = 1.33	0.721
AKI, n (%)	2,682 (80.35)	2,746 (82.29)	2,782 (83.34)	2,830 (84.78)	χ^2^ = 24.37	<0.001
Myocardial Infarction, n (%)	651 (19.50)	689 (20.65)	685 (20.52)	645 (19.32)	χ^2^ = 2.91	0.405
Congestive heart failure, n (%)	1035 (31.01)	1161 (34.79)	1217 (36.46)	1199 (35.92)	χ^2^ = 26.77	<0.001
Diabetes, n (%)	1254 (37.57)	1194 (35.78)	1119 (33.52)	1057 (31.67)	χ^2^ = 29.44	<0.001
Renal disease, n (%)	745 (22.32)	852 (25.53)	909 (27.23)	1012 (30.32)	χ^2^ = 57.52	<0.001
Invasive Mechanical Ventilation, n (%)	2,182 (65.37)	2,222 (66.59)	2,200 (65.91)	1950 (58.42)	χ^2^ = 62.84	<0.001
CRRT, n (%)	216 (6.47)	234 (7.01)	331 (9.92)	521 (15.61)	χ^2^ = 199.46	<0.001
Albumin use, n (%)	1133 (33.94)	1194 (35.78)	1150 (34.45)	1094 (32.77)	χ^2^ = 6.90	0.075
Glucocorticoid, n (%)	584 (17.50)	615 (18.43)	765 (22.92)	1071 (32.09)	χ^2^ = 253.59	<0.001
Apsiii	44.000 (32.0,60.0)	46.000 (33.0,61.0)	49.000 (37.0,64.0)	57.000 (44.0,74.0)	680.55	<0.001
Sapsii	37.000 (29.0,46.0)	37.000 (30.0,46.0)	39.000 (31.0,49.0)	42.000 (34.0,53.0)	332.10	<0.001
SOFA	6.000 (4.0,8.0)	6.000 (4.0,9.0)	7.000 (5.0,10.0)	8.000 (6.0,12.0)	779.25	<0.001
GCS	14.000 (9.0,15.0)	14.000 (9.0,15.0)	14.000 (9.0,15.0)	13.000 (8.0,14.0)	37.18	<0.001
LODS	5.000 (3.0,7.0)	5.000 (3.0,7.0)	5.000 (4.0,8.0)	6.000 (4.0,9.0)	379.94	<0.001
Charlson	4.000 (2.0,6.0)	5.000 (3.0,7.0)	5.000 (3.0,7.0)	5.000 (3.0,7.0)	135.96	<0.001
Meld	12.000 (9.0,19.7)	13.848 (10.0,21.0)	15.825 (10.0,23.2)	21.000 (13.9,28.0)	952.02	<0.001
Oasis	33.000 (28.0,39.0)	34.000 (29.0,39.0)	34.000 (29.0,40.0)	35.000 (29.0,42.0)	98.88	<0.001
RR (beats/min)	18.000 (15.0,22.0)	18.000 (15.0,23.0)	19.000 (16.0,24.0)	20.000 (17.0,25.0)	268.14	<0.001
MBP(mmHg)	81.000 (70.0,92.0)	80.500 (70.0,92.0)	79.000 (69.0,91.0)	78.000 (68.0,91.0)	25.42	<0.001
BMI	28.307 (24.4,33.4)	28.279 (24.4,33.2)	27.628 (24.1,32.9)	27.762 (24.0,32.9)	8.70	0.034*
OI	246.000 (154.3,350.0)	236.000 (148.5,344.0)	235.000 (142.0,347.0)	215.000 (134.8,318.0)	40.18	<0.001
Lac(mmol/L)	1.500 (1.1,2.2)	1.500 (1.1,2.1)	1.600 (1.1,2.5)	2.000 (1.3,3.5)	248.31	<0.001
WBC count (×109/L)	9.900 (6.9,13.9)	11.600 (8.2,15.9)	12.700 (8.8,17.1)	13.500 (8.7,19.4)	495.48	<0.001
NRBC count (×109/L)	0.700 (0.2,2.0)	0.500 (0.2,1.6)	0.500 (0.2,1.0)	0.700 (0.2,1.6)	6.58	0.087
Eosinophils count (×109/L)	0.100 (0.0,0.2)	0.068 (0.0,0.2)	0.032 (0.0,0.1)	0.010 (0.0,0.1)	1563.10	<0.001
Basophils count (×109/L)	0.030 (0.0,0.1)	0.028 (0.0,0.0)	0.020 (0.0,0.0)	0.010 (0.0,0.0)	786.54	<0.001
Monocytes count (×109/L)	0.495 (0.3,0.8)	0.529 (0.3,0.8)	0.556 (0.3,0.9)	0.520 (0.3,0.9)	33.96	<0.001
PLT count (×109/L)	229.00 (169.00,315.00)	192.00 (137.00,260.00)	171.00 (121.00,237.00)	129.00 (76.00,191.00)	1268.59	<0.001
MPV(fL)	8.70 (8.00,9.40)	9.00 (8.20,10.10)	9.60 (9.00,10.30)	10.20 (9.60,10.80)	16.29	<0.001
HCT (%)	31.800 (27.4,36.7)	31.300 (27.0,36.7)	31.500 (26.8,36.5)	31.100 (26.4,36.2)	20.06	<0.001
RDW (%)	14.400 (13.4,16.0)	14.400 (13.3,15.9)	14.700 (13.5,16.5)	15.400 (14.1,17.3)	434.08	<0.001
CRP (mg/L)	83.750 (17.4,161.8)	105.300 (23.7,176.6)	110.300 (47.9,193.4)	113.900 (56.0,214.8)	20.64	<0.001
Alb(g/dL)	3.100 (2.7,3.6)	3.100 (2.7,3.5)	3.000 (2.6,3.4)	2.900 (2.4,3.3)	120.47	<0.001
Cr (mg/dL)	1.000 (0.7,1.5)	1.000 (0.8,1.6)	1.100 (0.8,1.9)	1.400 (0.9,2.5)	448.74	<0.001
Bun (mg/dL)	19.000 (13.0,31.0)	20.000 (14.0,33.0)	22.000 (15.0,38.0)	30.000 (18.0,52.0)	596.56	<0.001
Calcium total (mg/dL)	8.400 (7.9,8.9)	8.300 (7.8,8.8)	8.200 (7.7,8.7)	8.100 (7.5,8.7)	233.11	<0.001
PT(s)	14.300 (12.7,16.6)	14.700 (12.9,17.3)	15.000 (13.1,18.2)	16.000 (13.4,21.0)	324.43	<0.001
APTT(s)	31.200 (27.4,37.9)	31.200 (27.4,38.4)	31.700 (27.5,39.0)	33.400 (28.4,42.5)	97.74	<0.001
INR	1.300 (1.1,1.5)	1.300 (1.2,1.6)	1.400 (1.2,1.7)	1.500 (1.2,1.9)	334.83	<0.001
FIB(mg/dL)	229.000 (179.0,329.0)	224.000 (173.0,320.0)	227.000 (169.0,360.0)	252.000 (152.0,444.8)	6.80	0.079
D-dimer (ng/mL)	1622.000 (695.0,4039.0)	1462.000 (799.0,4647.0)	2,217.000 (1001.0,5982.0)	3,692.000 (1331.8,7113.3)	19.90	<0.001
CKMB(ng/mL)	4.000 (2.0,10.0)	5.000 (3.0,14.0)	5.500 (3.0,15.0)	6.000 (3.0,14.0)	47.18	<0.001
ntprobnp (pg/ml)	3,290.000 (918.5,10145.5)	3,296.000 (1163.3,10313.5)	4,059.500 (1394.5,11793.8)	5754.000 (1647.5,15684.0)	17.53	0.001
ALT (U/L)	26.000 (15.5,51.0)	28.000 (16.0,61.0)	31.000 (18.0,73.0)	35.000 (19.0,85.0)	120.79	<0.001
AST (U/L)	36.000 (22.0,74.0)	42.500 (24.0,93.0)	48.000 (26.0,116.0)	56.500 (31.0,134.0)	201.88	<0.001
ALP(U/L)	85.000 (61.0,123.0)	82.000 (60.0,124.0)	89.000 (63.0,136.0)	95.000 (67.0,145.0)	76.29	<0.001
GGT (U/L)	89.000 (21.0,196.0)	65.000 (19.5,248.8)	110.000 (69.0,255.0)	101.500 (56.0,252.8)	5.34	0.149
TBIL (mg/dL)	0.500 (0.3,0.9)	0.600 (0.4,1.2)	0.700 (0.4,1.7)	1.100 (0.5,3.2)	697.83	<0.001
DBIL (mg/dL)	1.550 (0.6,4.0)	1.600 (0.5,4.0)	2.050 (0.8,4.4)	2.500 (1.2,6.0)	31.78	<0.001
LDH(U/L)	271.000 (200.0,419.0)	295.000 (213.0,458.0)	313.000 (224.0,486.5)	326.000 (232.3,509.8)	78.77	<0.001
Cl (mmol/L)	104.000 (100.0,107.0)	103.000 (99.0,107.0)	103.000 (99.0,107.0)	103.000 (98.0,107.0)	23.86	<0.001
glucose (mg/dL)	120.000 (99.0,161.0)	123.000 (102.0,165.0)	129.000 (104.0,173.0)	132.000 (104.0,179.0)	80.30	<0.001
K (mmol/L)	4.100 (3.7,4.5)	4.100 (3.8,4.6)	4.100 (3.7,4.6)	4.100 (3.7,4.7)	2.01	0.571
Na (mmol/L)	138.000 (136.0,141.0)	138.000 (135.0,141.0)	138.000 (135.0,140.3)	137.000 (134.0,141.0)	85.01	<0.001
TC (mg/dL)	153.000 (115.0,186.0)	147.000 (112.0,173.0)	123.000 (94.5,157.0)	119.000 (87.0,155.5)	38.12	<0.001
TG (mg/dL)	138.500 (90.8,225.5)	131.000 (90.0,224.5)	133.000 (88.0,222.5)	136.500 (88.0,240.3)	0.91	0.823
HDL (mg/dL)	38.000 (30.0,50.0)	39.000 (30.5,53.5)	37.000 (21.8,48.0)	34.500 (23.0,49.0)	7.03	0.071
LDL (mg/dL)	86.000 (55.0,115.5)	76.000 (54.0,100.0)	63.500 (43.0,89.3)	56.500 (33.3,80.8)	39.51	<0.001
NLR	3.673 (2.5,5.2)	6.835 (4.8,9.5)	11.352 (7.9,16.0)	23.339 (14.8,38.7)	7985.31	<0.001
PLR	122.814 (75.7,209.5)	145.418 (83.3,245.8)	188.247 (106.5,320.2)	252.730 (118.0,489.1)	979.50	<0.001

Patients with elevated NLPR exhibited a greater burden of comorbidities, with significantly higher rates of acute kidney injury, congestive heart failure, and chronic kidney disease in Q3 and Q4 (all P < 0.001). In contrast, the prevalence of diabetes decreased with increasing NLPR (P < 0.001), a finding that may reflect the complex interplay between metabolic status, immune response, and inflammatory dysregulation in sepsis. Additionally, disease severity scores, including APACHE III, SAPS II, and SOFA, increased progressively across quartiles (all P < 0.001), suggesting a direct correlation between NLPR and critical illness severity. Similarly, MELD, LODS, and Charlson Comorbidity Index scores were significantly higher in the upper quartiles (all P < 0.001), further reinforcing the association between NLPR and multi-organ dysfunction.

Inflammatory and coagulation markers varied significantly with NLPR levels. Higher quartiles were associated with progressively increased levels of CRP, lactate, and D-dimer (all P < 0.001), reflecting a heightened systemic inflammatory and prothrombotic state. Conversely, albumin levels declined with increasing NLPR (P < 0.001), consistent with worsening nutritional and inflammatory burdens in patients with more severe disease.

Total WBC count showed a significant upward trend across NLPR quartiles (P < 0.001), with the highest values observed in Q4. This increase aligns with an intensified inflammatory response, as WBC elevation is a hallmark of systemic infection. However, despite its long-standing role as an inflammatory marker in sepsis, WBC count alone lacks specificity in capturing the intricate immune-coagulation interactions that drive disease progression. In contrast, PLT count demonstrated an inverse association with NLPR (P < 0.001), progressively decreasing across quartiles. This decline is indicative of coagulopathy and microvascular dysfunction, both of which are well-recognized complications of sepsis-associated disseminated intravascular coagulation. However, platelet count alone does not sufficiently reflect the integrated inflammatory and immunological disturbances in sepsis. The requirement for intensive organ support also varied significantly across NLPR quartiles. The proportion of patients requiring CRRT increased markedly in Q3 and Q4 (P < 0.001), reflecting a greater degree of renal dysfunction in those with higher NLPR values. Interestingly, the need for invasive mechanical ventilation was lower in Q4 compared to Q1–Q3 (P < 0.001), which may suggest variations in clinical management strategies or distinct disease trajectories in this subgroup.

Collectively, these findings underscore that elevated NLPR is associated with a more pronounced inflammatory response, coagulation abnormalities, immune dysregulation, and multi-organ dysfunction. Compared with traditional markers such as WBC count and platelet count, NLPR provides a more comprehensive assessment of sepsis severity by integrating information on both inflammatory and coagulation pathways. The strong associations between NLPR and established disease severity scores further support its potential as a robust prognostic biomarker for risk stratification and individualized management in sepsis patients.

### 3.2 Survival analysis of NLPR levels across four patient groups

The analysis revealed a progressive increase in mortality rates across short-, mid-, and long-term periods with rising NLPR levels (see Table 2, P < 0.001). For short-term survival, the 28-day mortality rate in the Q4 group was 28.22%, significantly higher than the 12.64% observed in the Q1 group. A similar pattern was evident in mid-term survival, with the 90-day mortality rate reaching 36.82% in the Q4 group, markedly exceeding the 18.06% recorded in the Q1 group (P < 0.001). For long-term survival, the 365-day mortality rate climbed to 44.94% in the Q4 group, significantly surpassing the 25.58% in the Q1 group (P < 0.001). These results underscore NLPR as a robust and reliable predictor of mortality across all timeframes, reinforcing its potential utility in prognostic stratification.

**TABLE 2:** Cross-analysis of NLPR levels and survival rates among four patient groups.

Variables	Total (n = 13351)	1 (n = 3,338)	2 (n = 3,337)	3 (n = 3,338)	4 (n = 3,338)	Statistic	P
Mortality28, n (%)						χ^2^ = 343.44	<0.001
N	10901 (81.65)	2,916 (87.36)	2,887 (86.51)	2,702 (80.95)	2,396 (71.78)		
Y	2,450 (18.35)	422 (12.64)	450 (13.49)	636 (19.05)	942 (28.22)		
Mortality90, n (%)						χ^2^ = 388.05	<0.001
N	9996 (74.87)	2,735 (81.94)	2,685 (80.46)	2,467 (73.91)	2,109 (63.18)		
Y	3,355 (25.13)	603 (18.06)	652 (19.54)	871 (26.09)	1229 (36.82)		
Mortality365, n (%)						χ^2^ = 351.38	<0.001
N	8916 (66.78)	2,484 (74.42)	2,425 (72.67)	2,169 (64.98)	1838 (55.06)		
Y	4,435 (33.22)	854 (25.58)	912 (27.33)	1169 (35.02)	1500 (44.94)		

χ^2^: Chi-square test.

### 3.3 Independent predictive value of NLPR

Both univariate and multivariate Cox regression analyses demonstrated that NLPR is an independent predictor of mortality across all time points (see Table 3). After adjusting for confounding factors, patients in the Q4 group exhibited a significantly higher risk of 28-day mortality compared to the Q1 group (HR = 1.21, 95% CI: 1.07–1.36, P = 0.002). Similarly, the 90-day (HR = 1.23, 95% CI: 1.11–1.36, P < 0.001) and 365-day (HR = 1.19, 95% CI: 1.09–1.29, P < 0.001) mortality risks were markedly elevated in the Q4 group. Additionally, SOFA and SAPS II scores were significantly associated with mortality risk, underscoring the complementary role of NLPR in enhancing traditional scoring systems for optimized risk stratification.

**TABLE 3:** Analysis of cox proportional hazards regression models.

Variables	Univariate					Multivariate				
	β	S.E	Z	P	HR (95%CI)	β	S.E	Z	P	HR (95%CI)
28-day mortality										
Age	−0.00	0.00	−0.39	0.693	1.00 (1.00–1.00)					
Gender										
Male					1.00 (Reference)					
Female	0.05	0.04	1.22	0.224	1.05 (0.97–1.14)					
Sapsii	0.03	0.00	25.69	<0.001	1.03 (1.03–1.04)	0.02	0.00	11.26	<0.001	1.02 (1.01–1.02)
Sofa	0.13	0.00	30.40	<0.001	1.14 (1.13–1.15)	0.10	0.01	19.15	<0.001	1.10 (1.09–1.12)
BMI	0.01	0.00	2.06	0.040	1.01 (1.01–1.01)					
OI	−0.01	0.00	−3.54	<0.001	0.99 (0.99–0.99)					
Group										
Q1					1.00 (Reference)					1.00 (Reference)
Q2	0.07	0.07	0.96	0.336	1.07 (0.93–1.22)	0.04	0.07	0.60	0.547	1.04 (0.91–1.19)
Q3	0.25	0.06	3.90	<0.001	1.28 (1.13–1.45)	0.12	0.06	1.86	0.063	1.12 (0.99–1.27)
Q4	0.50	0.06	8.53	<0.001	1.65 (1.47–1.85)	0.19	0.06	3.16	0.002	1.21 (1.07–1.36)
90-day mortality										
Age	0.00	0.00	1.20	0.229	1.00 (1.00–1.00)					
Gender										
Male					1.00 (Reference)					
Female	0.02	0.03	0.50	0.614	1.02 (0.95–1.09)					
Sapsii	0.03	0.00	27.31	<0.001	1.03 (1.03–1.03)	0.02	0.00	12.07	<0.001	1.02 (1.01–1.02)
Sofa	0.12	0.00	32.06	<0.001	1.13 (1.12–1.14)	0.09	0.00	19.71	<0.001	1.09 (1.08–1.10)
BMI	0.00	0.00	0.44	0.657	1.00 (1.00–1.01)					
OI	−0.01	0.00	−3.62	<0.001	0.99 (0.99–0.99)					
Group										
Q1					1.00 (Reference)					1.00 (Reference)
Q2	0.09	0.06	1.56	0.118	1.09 (0.98–1.22)	0.06	0.06	1.15	0.252	1.07 (0.95–1.19)
Q3	0.23	0.05	4.38	<0.001	1.26 (1.14–1.40)	0.12	0.05	2.25	0.025	1.13 (1.02–1.25)
Q4	0.48	0.05	9.55	<0.001	1.61 (1.46–1.78)	0.21	0.05	4.07	<0.001	1.23 (1.11–1.36)
365-day mortality										
Age	0.01	0.00	2.65	0.008	1.01 (1.01–1.01)					
Gender										
Male					1.00 (Reference)					
Female	−0.02	0.03	−0.53	0.598	0.98 (0.93–1.04)					
Sapsii	0.03	0.00	26.58	<0.001	1.03 (1.03–1.03)	0.01	0.00	11.91	<0.001	1.01 (1.01–1.02)
Sofa	0.11	0.00	30.77	<0.001	1.11 (1.10–1.12)	0.08	0.00	18.55	<0.001	1.08 (1.07–1.09)
BMI	0.00	0.00	0.01	0.991	1.00 (1.00–1.00)					
OI	−0.01	0.00	−2.53	0.011	0.99 (0.99–0.99)					
Group										
Q1					1.00 (Reference)					1.00 (Reference)
Q2	0.10	0.05	2.06	0.039	1.10 (1.01–1.21)	0.08	0.05	1.58	0.113	1.08 (0.98–1.18)
Q3	0.22	0.05	4.98	<0.001	1.25 (1.15–1.37)	0.14	0.05	3.02	0.003	1.15 (1.05–1.25)
Q4	0.39	0.04	9.06	<0.001	1.48 (1.36–1.61)	0.17	0.04	3.91	<0.001	1.19 (1.09–1.29)

HR, hazard ratio; CI, confidence interval.

### 3.4 Kaplan-Meier survival curve analysis

**FIGURE 2:**
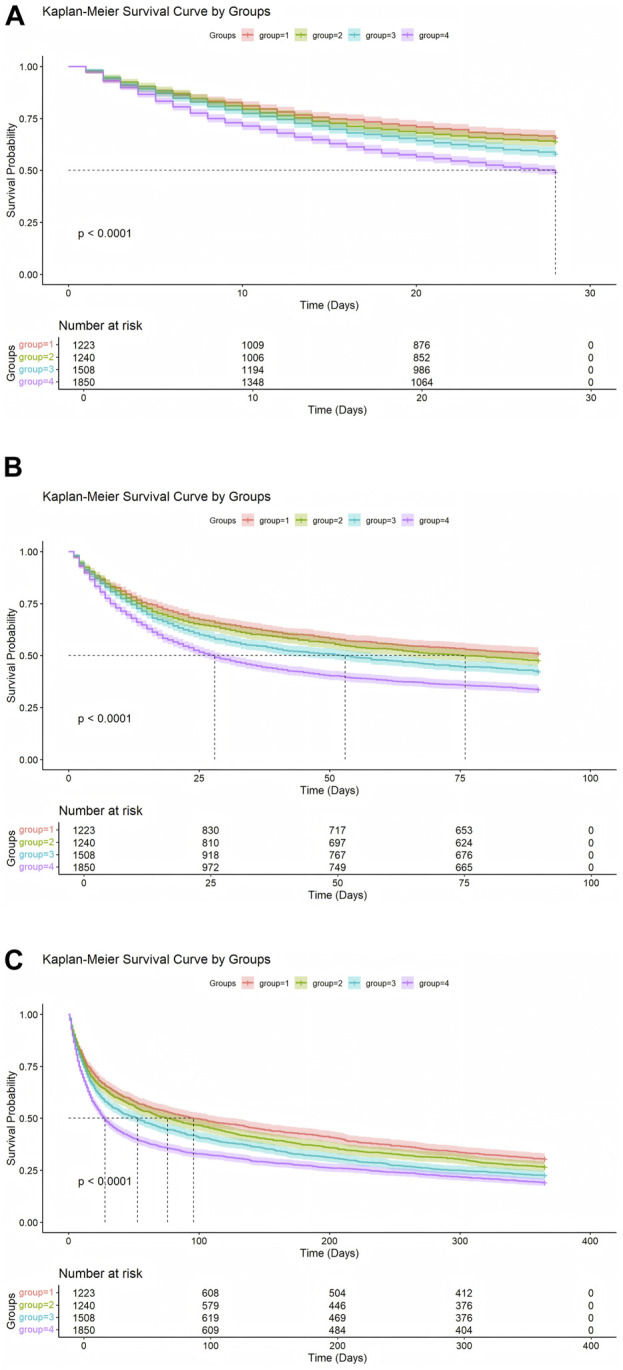
**(A)** Kaplan-Meier Analysis of 28-Day Survival probabilities. **(B)** Kaplan-Meier Analysis of 90-Day Survival probabilities. **(C)** Kaplan-Meier Analysis of 365-Day Survival probabilities.

Kaplan-Meier survival analysis (Figure 2) revealed significant differences in survival probabilities among the NLPR groups (Log-rank test, P < 0.001). Across all time points, patients in the Q1 group consistently exhibited the highest survival rates, while those in the Q4 group showed the lowest survival probabilities. A pronounced negative correlation was observed between NLPR levels and survival probabilities at 28-day, 90-day, and 365-day follow-ups. These findings further underscore the practical utility of NLPR in clinical risk stratification and highlight its potential role as a prognostic biomarker.

### 3.5 Restricted cubic spline analysis

**FIGURE 3:**
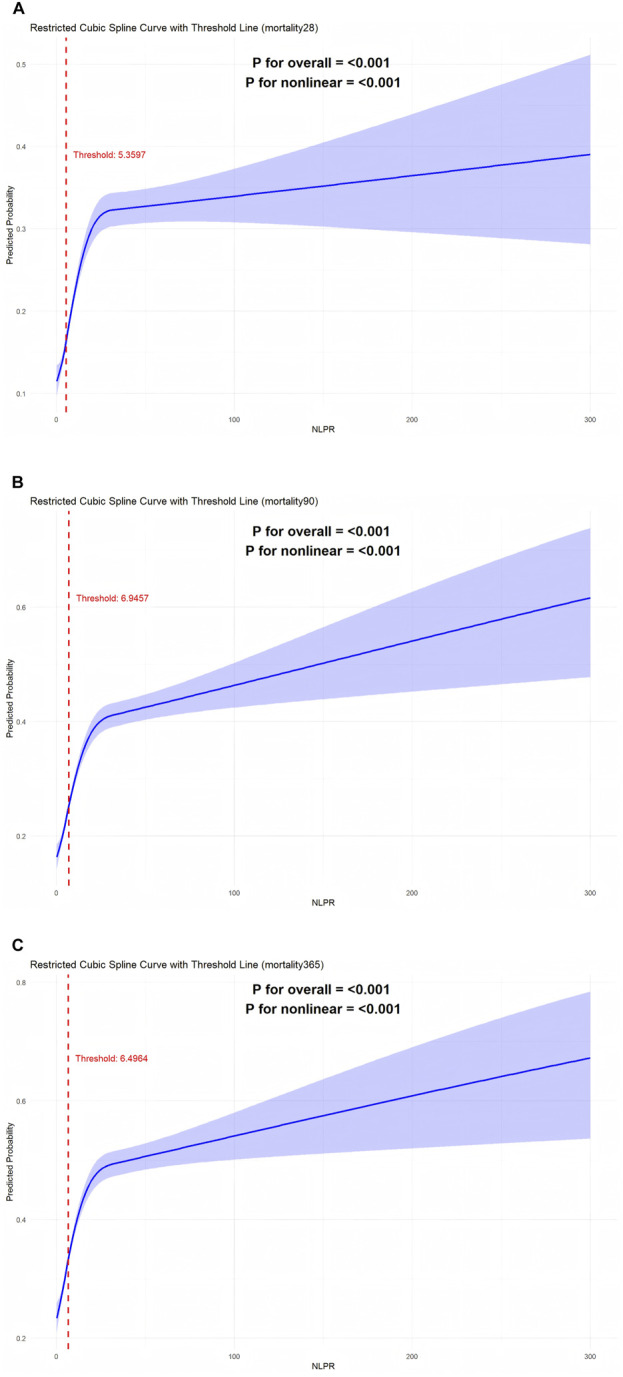
**(A)** RCS curve analysis of 28-day survival rate. **(B)** Rcs curve analysis of 90-day survival rate. **(C)** Rcs curve analysis of 365-day survival rate.

In this study, a restricted cubic spline analysis was conducted to investigate the nonlinear relationship between the NLPR and mortality, while adjusting for multiple confounding factors, including age,gender,BMI,OI, and SOFA score (Figure 3). For 28-day all-cause mortality, the RCS analysis (Figure 3A) revealed a nonlinear association, with a turning point at NLPR = 5.3597. Below this threshold, the risk of mortality increased gradually with minor elevations in NLPR; however, once NLPR surpassed this value, the mortality risk escalated sharply, suggesting that elevated NLPR values may indicate severe dysregulation of inflammatory and coagulation states in patients. In the analysis of 90-day mortality (Figure 3B), the turning point on the RCS curve was identified at NLPR = 6.9457. Beyond this threshold, a significant increase in 90-day mortality was observed, indicating that elevated NLPR values may serve as a critical warning indicator of poor mid-term prognosis. For 365-day mortality (Figure 3C), the turning point was determined to be NLPR = 6.4964, highlighting the predictive effect of NLPR on long-term survival. Higher NLPR values were associated with persistent inflammation, coagulation dysfunction, and the cumulative impact of multi-organ failure. Overall, the RCS analysis clearly illustrated the nonlinear relationship between NLPR and mortality, identifying critical thresholds for each time point. These findings not only provide robust evidence for NLPR as a prognostic biomarker in sepsis patients but also support its potential utility in clinical risk stratification and personalized therapeutic decision-making.

### 3.6 Subgroup analysis: impact of NLPR on mortality

**FIGURE 4:**
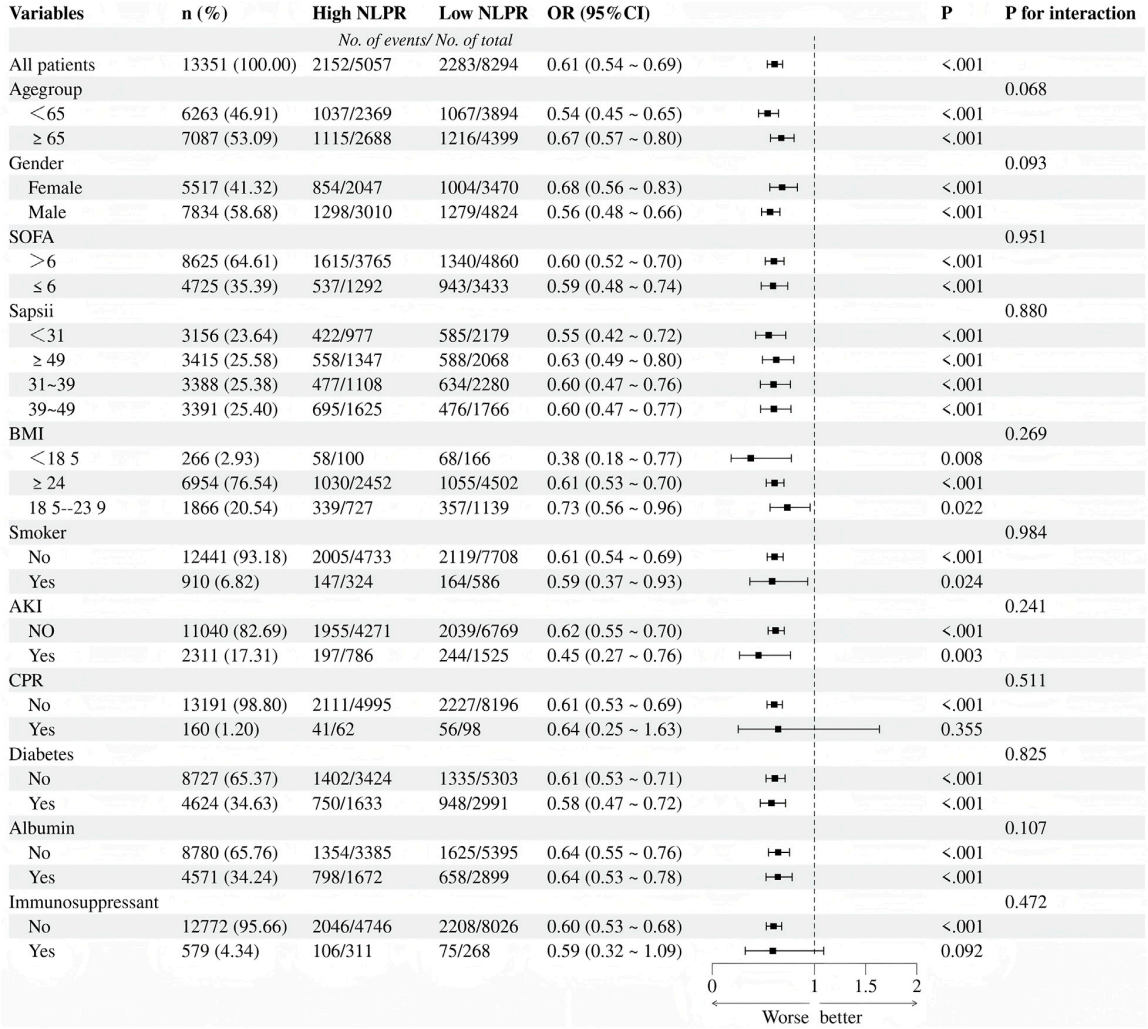
Adjusted forest plot of subgroup analysis.

In the subgroup analysis (Figure 4), elevated NLPR levels were significantly associated with an increased risk of mortality (OR = 1.64, 95%CI:1.45–1.86, P < 0.001), and this association demonstrated strong consistency across various clinical subgroups. When stratified by age, the impact of NLPR on mortality risk was more pronounced in patients aged ≤65 years (OR = 1.85, 95%CI:1.55–2.21, P < 0.001), suggesting that younger patients may experience greater pathophysiological effects from inflammation and coagulation dysregulation. The gender subgroup analysis revealed that high NLPR was significantly associated with an increased mortality risk in both males (OR = 1.77, 95%CI:1.51–2.07, P < 0.001) and females (OR = 1.46, 95%CI:1.20–1.78,P < 0.001), with no significant interaction effect between gender and NLPR (P for interaction = 0.093). Further stratification by SOFA and SAPS II scores confirmed this trend: regardless of disease severity, elevated NLPR was consistently linked to a higher mortality risk (P < 0.001 for all). Notably, patients with a BMI <18 exhibited a particularly significant increase in mortality risk (OR = 2.65, 95%CI:1.29–5.42, P = 0.008), highlighting the vulnerability of malnourished individuals to inflammation and coagulation disturbances. Additionally, patients with AKI showed a markedly elevated risk of death (OR = 2.21, 95%CI: 1.31–3.75, P = 0.003), further emphasizing the critical role of multi-organ dysfunction in patients with high NLPR.

The subgroup analysis emphasizes the strong predictive performance of NLPR for mortality across various clinical populations, with an even more pronounced risk amplification noted in specific high-risk subgroups, such as malnourished patients and those with acute kidney injury. These findings underscore the utility of NLPR as a crucial prognostic biomarker in sepsis, providing valuable insights for the identification of high-risk populations and the formulation of targeted, individualized treatment strategies.

## 4 Discussion

Sepsis is characterized by a dysregulated host response to infection, often resulting in multi-organ dysfunction and, in severe cases, progressing to septic shock. Despite advancements in treatment strategies that focus on infection control and organ support, sepsis remains a major global health burden, with high mortality rates, particularly among patients with multiple organ dysfunction syndrome (Giamarellos-Bourboulis et al., 2024). Within the complex and dynamic pathophysiology of sepsis, the interplay between the inflammatory response and the coagulation system plays a central role (Bauer et al., 2020). In the early stages of infection, the host initiates an inflammatory cascade to activate the immune system and control the spread of pathogens. However, excessive inflammatory responses may lead to a cytokine storm, resulting in endothelial damage and platelet activation, which disrupts coagulation and exacerbates microcirculatory dysfunction. As the disease progresses, some patients may experience immune exhaustion and coagulation suppression, rendering infections uncontrollable and leading to multi-organ failure (Bomrah et al., 2024; Slim et al., 2024; Harhay et al., 2014).

To address the need for a simple yet effective biomarker that integrates inflammation and coagulation dysfunction, this study focuses on the prognostic value of the NLPR in sepsis patients. The findings suggest that NLPR serves as a comprehensive and reliable biomarker for risk stratification, complementing traditional scoring systems such as SOFA and SAPS II. NLPR was significantly associated with short-term (28-day), mid-term (90-day), and long-term (365-day) mortality, providing a valuable tool for individualized treatment decision-making.

### 4.1 NLPR and patient heterogeneity

This study revealed significant baseline heterogeneity among sepsis patients categorized by NLPR quartiles. Patients in the higher NLPR groups (Q3 and Q4) were older, predominantly male, and more frequently widowed or single. Socioeconomic factors, lifestyle differences, and access to healthcare may contribute to these disparities, potentially influencing sepsis outcomes (Singer et al., 2016; Cuenca et al., 2022; Balamuth et al., 2022). Prior studies indicate that social isolation and lack of support networks are associated with worse prognoses in critically ill patients, compounding disease burden (Leligdowicz and Matthay, 2019).

Additionally, higher NLPR quartiles were associated with increased comorbidities, including acute kidney injury, congestive heart failure, and chronic kidney disease. This pattern suggests that elevated NLPR reflects a state of heightened inflammation, impaired immune response, and multi-organ dysfunction, all of which are key determinants of sepsis severity. The concurrent elevation in APACHE II, SAPS II, and SOFA scores in high NLPR groups further underscores the necessity for aggressive organ support and precise management strategies in this cohort.

### 4.2 Prognostic value of NLPR for survival in sepsis patients

This study confirms NLPR as a robust prognostic biomarker for sepsis, showing strong associations with key inflammatory and coagulation markers, including CRP, lactate, and D-dimer, as well as decreased albumin and lymphocyte counts. Elevated CRP reflects sustained systemic inflammation, while increased D-dimer suggests excessive activation of coagulation and fibrinolysis, often linked to microcirculatory dysfunction and endothelial damage (van Amstel et al., 2023; Giustozzi et al., 2021; Bode et al., 2023; Molema et al., 2022). Hypoalbuminemia, indicative of severe systemic inflammation and nutritional decline, has also been associated with increased mortality in sepsis patients (Sinha et al., 2023; Ruot et al., 2000).

Notably, Kaplan-Meier survival analysis demonstrated a stepwise increase in mortality across NLPR quartiles, with significantly higher 28-day (28.22%), 90-day (36.82%), and 365-day (44.94%) mortality rates in the highest quartile (Q4) compared to the lowest quartile (Q1) (all P < 0.001). This further supports NLPR as a reliable indicator of disease progression and survival risk. To further evaluate its predictive performance, we compared NLPR with traditional inflammatory and coagulation markers, namely WBC and platelet count.

### 4.3 Comparison of NLPR with traditional inflammatory and coagulation markers

While WBC and platelet counts have been widely used in clinical assessments of sepsis, their standalone predictive accuracy is limited due to their inability to capture the dynamic immune-coagulation interplay. Our study provides a direct comparison, demonstrating the superior prognostic value of NLPR.

(1) WBC count: While significantly elevated in high NLPR groups (P < 0.001), suggesting an intensified inflammatory response, WBC alone had limited predictive power for mortality when assessed independently.
(2) PLT count: A reduction in platelet count is a well-recognized indicator of coagulopathy and microvascular dysfunction in sepsis. However,PLT alone lacks the ability to fully reflect immune-inflammatory dysregulation, making it less effective as a single prognostic marker.

Multivariate Cox regression analysis confirmed that only NLPR retained independent predictive significance for mortality (P < 0.001), even after adjusting for age, gender, BMI, and SOFA score, whereas WBC and platelet count lost their statistical significance. This underscores NLPR’s advantage in integrating inflammation, coagulation dysfunction, and immune suppression into a single risk-stratification tool.

Additionally, subgroup analyses demonstrated that NLPR consistently outperformed WBC and platelet count across different patient subgroups, particularly in younger patients, those with a low BMI, and those with acute kidney injury. These findings highlight the clinical potential of NLPR as a more refined biomarker that captures the multifaceted pathophysiology of sepsis, supporting its use as a practical, real-time prognostic tool.

In addition to WBC and PLT counts, we analyzed mean platelet volume (MPV) across the four NLPR quartiles to further explore its association with inflammation, coagulation dysfunction, and disease severity in sepsis patients. Our findings revealed a progressive increase in MPV with rising NLPR levels, suggesting enhanced platelet activation in patients with higher inflammatory and coagulation burdens. This trend aligns with previous studies indicating that larger platelets exhibit greater metabolic and enzymatic activity, contributing to a prothrombotic state and exacerbating microvascular dysfunction in critically ill patients. Despite the observed decline in platelet count with increasing NLPR, the concomitant rise in MPV suggests a compensatory response to systemic inflammation and coagulation disturbances. This underscores the critical interplay between platelet function and sepsis pathophysiology, reinforcing the notion that platelet indices provide valuable insights beyond absolute PLT counts. Given the strong association between MPV and adverse outcomes in sepsis, incorporating MPV into future risk assessment models alongside NLPR may enhance prognostic accuracy and guide individualized therapeutic strategies.

### 4.4 Nonlinear association between NLPR and mortality and its clinical implications

Restricted cubic spline analysis further elucidated a nonlinear relationship between NLPR and mortality, providing novel insights into its clinical utility as a prognostic biomarker. The analysis identified critical inflection points for NLPR: 5.3597 for 28-day all-cause mortality, 6.9457 for 90-day mortality, and 6.4964 for 365-day mortality. The results demonstrated that when NLPR values remained below these thresholds, the risk of mortality increased gradually with slight elevations in NLPR. However, once NLPR exceeded these thresholds, the risk of mortality escalated steeply. This nonlinear association underscores the significance of NLPR in sepsis risk stratification, aligning with prior studies that emphasize the role of inflammation and coagulation dysregulation in disease progression (Shankar-Hari et al., 2024).

This finding carries important clinical implications. First, the identified NLPR thresholds provide clear reference standards for the early identification of high-risk patients. For instance, when NLPR approaches or exceeds 6.5, it may indicate significant exacerbation of inflammatory and coagulation disorders (Rudd et al., 2020). At this juncture, prompt interventions, such as intensified anti-inflammatory therapies and coagulation regulation, could substantially improve patient outcomes. Second, compared to traditional scoring systems like SOFA and APACHE II, NLPR offers advantages in simplicity, ease of operation, and real-time monitoring, making it particularly effective for rapidly assessing disease severity and adjusting treatment strategies. These findings highlight NLPR’s potential to enhance clinical decision-making, offering a practical tool for optimizing risk stratification and guiding timely, individualized interventions in sepsis management.

### 4.5 Subgroup analysis: precision identification of high-risk populations

Subgroup analysis further validated the broad applicability of the NLPR across various patient populations while highlighting a pronounced amplification of risk in certain high-risk groups. Among younger patients (aged≤65 years), the NLPR exhibited particularly strong predictive performance for mortality, suggesting that the pathological effects of inflammation and coagulation abnormalities may be more pronounced in this cohort. Additionally, patients with a low BMI (BMI<18) demonstrated a significantly elevated risk of mortality, indicating a potential link between malnutrition and systemic inflammatory burden. Furthermore, in patients with acute kidney injury, the association between NLPR and mortality risk was particularly striking, underscoring the critical role of multi-organ dysfunction in the prognosis of patients with elevated NLPR levels. Gender-specific analysis revealed consistent predictive performance of NLPR in both males and females, indicating its universal applicability across genders. These findings not only confirm the robustness of NLPR across various subgroups but also provide strong evidence of its utility in identifying high-risk patients. By facilitating precise stratification, NLPR serves as a valuable tool for guiding targeted interventions and enhancing outcomes in vulnerable populations.

Based on these findings, the potential applications of the NLPR in the management of sepsis warrant further exploration. First, NLPR could serve as a valuable supplement to existing prognostic tools, aiding in risk stratification and the early identification of high-risk patients. Second, informed by the critical thresholds identified in ROC analysis, NLPR may be utilized for dynamic monitoring of disease progression, providing real-time guidance for clinical decision-making. For instance, in patients with significantly elevated NLPR, the early initiation of anti-inflammatory and anticoagulant therapies could improve outcomes. Additionally, integrating NLPR into electronic health record systems for real-time risk assessment through big data analytics could enhance the efficiency and effectiveness of sepsis management.

Although our study showed that NLPR is useful in clinical practice, it still has some limitations. First, this was a retrospective study, so it might have selection and information bias. Future prospective studies are needed to confirm these findings. Second, this study used data from only one center, which means the results, especially the cutoff values for NLPR, should be checked in multicenter studies with different patient populations. Another limitation is that we analyzed only all-cause mortality, because detailed causes of death (sepsis-related or unrelated) were not available from the MIMIC-IV database. This makes it important to conduct future prospective studies that can clearly identify causes of death. Doing so would provide a better understanding of how NLPR can predict outcomes in patients with sepsis.

## 5 Conclusion

This study comprehensively explores the clinical value of the NLPR in patients with sepsis, demonstrating its significant association with short-term (28-day), mid-term (90-day), and long-term (365-day) mortality. By integrating inflammatory and coagulation data, NLPR accurately reflects the core pathological processes underlying sepsis. Kaplan-Meier survival analysis and Cox regression models validate its reliability as an independent prognostic marker, while RCS analysis reveals the nonlinear relationship between NLPR and mortality, providing a foundation for the early identification and intervention of high-risk patients. Furthermore, subgroup analysis confirms the robust predictive performance of NLPR across diverse patient characteristics, particularly among those with multi-organ dysfunction. This study highlights the potential of NLPR for sepsis risk stratification and individualized therapy, offering a novel perspective for optimizing clinical management and improving patient outcomes.

## Data Availability

The original contributions presented in the study are included in the article/supplementary material, further inquiries can be directed to the corresponding author.
